# Identification of Ixodid Tick-Specific Aquaporin-1 Potential Anti-tick Vaccine Epitopes: An *in-silico* Analysis

**DOI:** 10.3389/fbioe.2019.00236

**Published:** 2019-09-26

**Authors:** Christian Ndekezi, Joseph Nkamwesiga, Sylvester Ochwo, Magambo Phillip Kimuda, Frank Norbert Mwiine, Robert Tweyongyere, Wilson Amanyire, Dennis Muhanguzi

**Affiliations:** ^1^School of Biosecurity, Biotechnical and Laboratory Science, College of Veterinary Medicine Animal Resources and Biosecurity, Makerere University, Kampala, Uganda; ^2^Research Unit in Bioinformatics, Department of Biochemistry and Microbiology, Rhodes University, Grahamstown, South Africa; ^3^School of Veterinary Medicine and Animal Resources, College of Veterinary Medicine Animal Resources and Biosecurity, Makerere University, Kampala, Uganda; ^4^Makerere University/Uganda Virus Research Institute Centre of Excellence in Infection and Immunity Research and Training, Entebbe, Uganda

**Keywords:** antigenicity, aquaporin-1 protein, tick control, peptide motifs, cattle

## Abstract

Ticks are arthropod vectors of pathogens of both Veterinary and Public health importance. Acaricide application, which is currently the mainstay of tick control, is hampered by high cost, the need for regular application and a selection of multi-acaricide resistant tick populations. In light of this, future tick control approaches are poised to rely on the integration of rational acaricide application and other methods, such as vaccination. To contribute to systematic research-guided efforts to produce anti-tick vaccines, we carried out an *in-silico* analysis of tick aquaporin-1 (AQP1) protein in order to identify tick-specific AQP1 peptide motifs that can be used in future peptide anti-tick vaccine development. We carried out multiple sequence alignment (MSA), motif analysis, homology modeling, and structural analysis to identify tick-specific AQP1 peptide motifs. BepiPred, Chou and Fasman-Turn, Karplus and Schulz Flexibility, and Parker-Hydrophilicity prediction models were used to predict these motifs' potential to induce B cell mediated immune responses. The tick AQP1 (GenBankID: QDO67142.1) protein was largely similar to the bovine AQP1 (PDB:1J4N) (23 % sequence similarity; Structural superimposition of the homology model and 14JN homotetramers gave an RMSD = 3.269 while superimposition of a single chain gave an RMSD = 1.475). Tick and bovine AQP1 transmembrane domains were largely similar while their extracellular and cytoplasmic domain loops showed variation. Two tick-specific AQP1 peptide motifs; M7 (residues 106–125, *p* = 5.4e-25), and M8 (residues 85–104, *p* = 3.3e-24) were identified. These two motifs are located on the extra-cellular AQP1 domain. Motifs; M7 and M8 showed the highest Parker-Hydrophilicity prediction immunogenicity scores of 1.784 and 1.536, respectively. These two motifs can be a good starting point for the development of potential tick AQP1 peptide-based anti-tick vaccines. Further analyses such as molecular dynamics, *in vitro* assays, and *in vivo* immunization assays are required to validate these findings.

## Background

**Figure 1:**
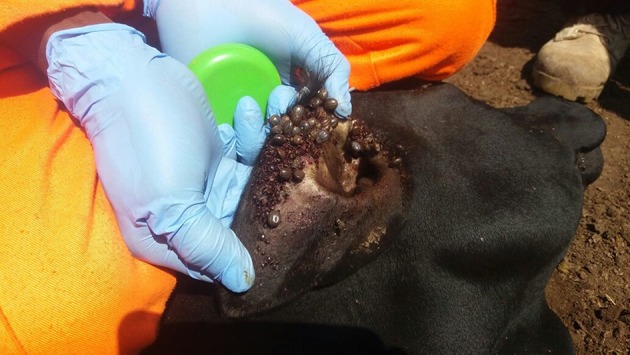
Heavily tick-infested short horn East African Zebu heifer from Serere District, South-eastern Uganda.

Ticks are arthropod vectors of pathogens of both Veterinary and Public health importance in tropical and sub-tropical regions of the world (Jongejan and Uilenberg, 2004; Wikel, 2018). Ticks are associated with both direct and indirect constraints to livestock health and production (Jongejan and Uilenberg, 2004; Wikel, 2018). Heavy tick infestation (Figure 1) is often associated with direct constraints to livestock production including blood loss (anemia), discomfort, skin-and-hide quality loss, and tick paralysis (Eskezia and Desta, 2016). Their indirect constraints to livestock production and health relate to transmission of diseases of veterinary importance including: anaplasmosis (*Anaplasma* spp.), babesiosis (*Babesia* spp.), theileriosis (*Theileria* spp.), and heartwater (*Cowdria ruminatium*) (Jongejan and Uilenberg, 2004). In addition, ticks transmit zoonotic pathogens belonging to *Babesia* spp., *Borrelia* spp., *Rickettsia* spp., *Ehrlichia* spp., *Francisella tularensis, Coxiella burnetii*, and viruses such as Nairovirus (*Bunyaviridae*) that causes Crimean-Congo Hemorrhagic Fever (CCHF) and tick-borne encephalitis virus (*Flaviviridae*) (Sambri et al., 2004; Telmadarraiy et al., 2015).

In order to reduce the impact of ticks on livestock health and production as well as withdraw their negative public health effects, acaricides, tick hand-picking, animal movement control, and vaccination are often applied to control ticks (Chizyuka and Mulilo, 1990; Jongejan, 1999). Acaricides are the most effective tick and tick-borne diseases (TBDs) control measure (Domingos et al., 2013). However, the ever increasing cost of acaricides in less developed countries, which are the most affected by ticks and TBDs, implies that acaricides cannot be regularly applied for tick control (Chizyuka and Mulilo, 1990). Moreover, acaricide overuse and misuse are associated with a selection of multi-acaricide resistant tick populations (Abbas et al., 2014); a problem that has been on the rise in Uganda (Vudriko et al., 2016). In light of this, future tick control strategies will have to depend on the integration of economically effective acaricide application, vaccination, breeding livestock for tick resistance, and other available tick control methods such as controlled animal movements (Ghosh and Azhahianambi, 2007). To date, anti-tick vaccine development has been slow. For example, the only commercially available recombinant vaccine against *R. microplus* was developed more than 2 decades ago (Rodríguez et al., 1995; Parizi et al., 2012). There is, therefore, a need to rekindle systematic research-guided efforts to evaluate crucial tick proteins and biological pathways for vaccine development, hence the motivation to evaluate tick specific AQP1 peptide motifs.

Tick aquaporins belong to the Membrane Intrinsic Protein (MIP) superfamily which are known to play a key role in the transportation of water, glycerol and urea across the cell membrane. MIP has three subfamilies; classical aquaporin (cAQP), aquaglyceroporin, and Super aquaporin (S-aquaporin). The major difference between the three subfamilies is in their sequence signature motifs around the protein pore. Classical aquaporins allow only the passage of water while aquaglyceroporins conduct glycerol, water, and small uncharged solutes. On the other hand, the role of S-aquaporin is currently not well known but it is believed to be involved in cellular differentiation, apoptosis, organogenesis, mating, and intercellular communication; activities that are important in multicellular organism (Ishibashi et al., 2011). There are two types of tick aquaporins. Both Aquaporins (AQP1 and AQP2) help in concentrating blood meals by facilitating the excretion of excess water back into the host through the salivary glands (Knepper, 1994; Ball et al., 2009). AQP1 is expressed in tissues such as the gut, rectal sac, and most abundantly in the tick salivary glands while AQP2 is only expressed in the salivary glands (Campbell et al., 2010; Hussein et al., 2015). Tick AQP1 recombinant antigens have previously been shown to be effective against different *I. ricinus* and *R. microplus* stages, highlighting the potential of using AQP1 as a candidate antigen for anti-tick vaccine development (Guerrero et al., 2014; Contreras and de la Fuente, 2017). It is against this background that tick AQP1 is localized in more tissues and is a more efficient water channel than tick AQP2 that we chose to assess tick AQP1 peptide motifs and not those of tick AQP2.

The development of an AQP1-derived anti-tick vaccine remains a challenge mostly due to the high sequence and structural similarities between the tick and host (humans and Bovine) AQP1 orthologs (Guerrero et al., 2014). However, this can potentially be overcome by identifying tick specific immunogenic regions within the AQP1 protein that will allow for a more selective immunogenic response. Furthermore, tick species isolated from different tick endemic regions are required to assess the conservation of these tick AQP1 immunogenic regions. In the study herein, we analyzed tick AQP1 from *Ixodid* ticks isolated from three different agro-ecological zones in Uganda and bovine AQP1 proteins derived from the Protein Data Bank (PDB) in order to identify tick-specific AQP1-peptide motifs (Berman et al., 2000). The tick-specific AQP1 peptide motifs were mapped onto the modeled 3-dimensional (3D) protein structure and further analyzed to determine their B cell stimulation potential. These results present peptides that might be of value in the development of potential anti-tick AQP1 peptide-based vaccines.

## Methods

### Study Design

**Figure 2:**
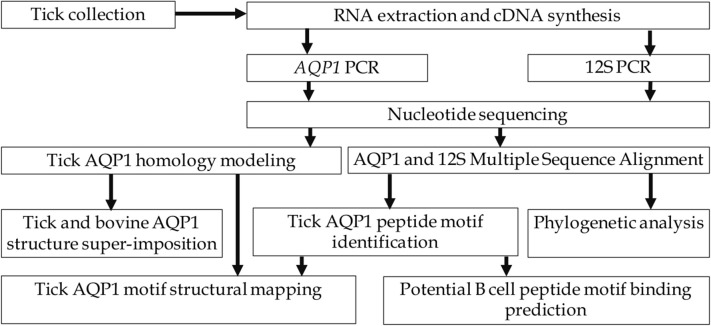
Study workflow.

This study involved identification and confirmation of representative tick species collected from cattle sampled from districts along the main cattle keeping agro-ecological zones of Uganda. The *AQP1* gene of these ticks was amplified by PCR and confirmed by Sanger sequencing. We used Multiple Sequence Alignment (MSA), motif analysis, homology modeling and structural analysis to identify tick-specific AQP1 peptide motifs. We then used Immune Epitope Database Analysis Resource (IEDB) to assess the immunogenicity of the tick-specific AQP1 peptide motifs (Figure 2).

### Study Area Description

**Figure 3:**
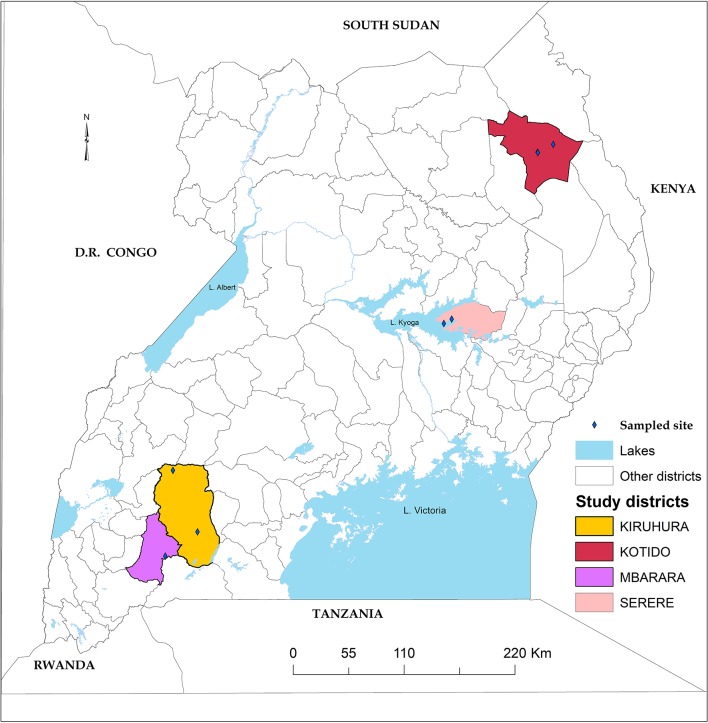
Study area. Four study districts highlighted with black boarders and colored fill.

Ticks were collected from cattle in four districts of Uganda, namely Serere (south-eastern Uganda), Kotido (north-eastern Uganda), Mbarara, and Kiruhuura (south-western Uganda) (Figure 3). The choice of these four districts was 2-fold; they represent the main cattle keeping Agro-ecological zones and have the highest tick burden in the country (MAAIF and UBOS, 2008; Kabi et al., 2014; Vudriko et al., 2016).

### Tick Collection, Preservation, and Morphological Tick Identification

Cattle in each village were gathered at a central cattle holding ground where the sampled cattle were physically restrained before a half body tick collection was carried out. Ticks were collected from each of the five predilection sites, namely (i) the inner and outer fore-legs, (ii) hind-legs and abdomen, (iii) tail and anal areas, (iv) head and neck, (v) lateral and dorsal areas, shoulders to tail base and ears. Collected tick specimens were put in different customized tick collection containers covered with gauze. The tick specimens were transferred to Makerere University, College of Veterinary Medicine, Animal Resources and Bio-security, Molecular Biology Laboratory for morphological and molecular identification to species level (Labruna et al., 2009; Farkas et al., 2013; Walker et al., 2014).

### RNA Extraction and cDNA Synthesis

Total RNA from homogenized tick samples was extracted using GE Healthcare RNA extraction kit (Chicago, USA). Complementary DNA (cDNA) was synthesized using a GoScript Reverse Transcriptase (Promega, Madison, Wisconsin, USA). Briefly, a mixture of 1 μl of RNA, 5 μl of poly T primers and 14 μl of nuclease-free water were preheated at 70°C for 10 min. The mixture was then placed on ice pending further steps. The enzyme mix (1 × first strand buffer, 4 μl of MgCl_2_, 2 mM dNTP, 0.51 μl of SUPERase inhibitor, 1 μl of superscript II, 5 μl of the RNA mix, and RNase-free water) was then incubated in a thermocycler according to the manufacturer's cycling conditions; one cycle of 10 min at 25°C, 45 min at 37°C, 45 min at 42°C, followed by 15 min at 70°C. The cDNA was used immediately or stored at −20°C until it was needed for further analysis.

### PCR Amplification of 12S rRNA and Tick *AQP1* Genes

Tick species were confirmed by PCR and Sanger sequencing of 12S ribosomal RNA (12S rRNA) as previously described by Labruna et al. (2009). The 12S PCR was carried out using a single pair of primers (12S-F-AAACTAGGATTAGATACCCT) and (12SR-AATGAGAGCGACGGGCGATGT) that amplify a 300 bp fragment of the 12S ribosomal RNA gene. Briefly, PCR reactions were prepared in 50 μl final reaction volumes that each contained 1.56 U *Taq* DNA polymerase (New England Biolabs, USA), 1 × standard buffer, 0.25 mM of each dNTP, 0.25 mM of each forward and reverse primer, 1.25 mM MgCl_2_, PCR-grade water and 5 μl of the cDNA template. The 12S rRNA and tick *AQP1* gene PCRs were completed in an S1000 Thermal Cycler (BIO-RAD, California, USA) with an initial denaturation of 94°C for 5 min followed by 30 cycles of 94°C for 30 s, 52°C for 45 s, 72°C for 45 s and a final extension of 72°C for 7 min.

We designed a single pair of primers (AQP1-1F- 5′GCGTGAAGATCAAGAACGCC3′ and AQP1-1R 5′GCCAATTGGAATCGAGGTGC3′) to amplify an 842 bp fragment spanning the entire tick *AQP1* gene. The tick *AQP1* PCR reactions were performed in an S1000 Thermal Cycler in 50 μl final reaction volume containing 1 × standard buffer, 0.25 mM of each dNTP, 0.25 mM each of forward and reverse primers, 1.56 U *Taq* DNA polymerase, 1.25 mM MgCl_2_, 32.18 μl of PCR-grade water and 5 μl of the cDNA template. The thermocycling conditions were; 95°C for 5 min, followed by 30 cycles of 95°C for 30 s, 50°C for 30 s, and 72°C for 1 min, and a final extension step at 72°C for 10 min.

### *AQP1*, and 12S Gene Amplicon Analysis and Sequencing

All PCR amplicons were resolved on 2% agarose (Bioline, Memphis, USA) gels stained with GelRed® Nucleic Acid Gel Stain (Biotium, California, USA) and visualized on an ultraviolet transilluminator (Wagtech International, Thatcham, UK) for fragment size determination. The amplicons were sized against a 50 bp DNA molecular ladder (Bioline, Memphis, USA). PCR products were purified from agarose gels using QIAquick® PCR purification kit (Hilden, Germany) according to manufacturer's instructions prior to sequencing. The PCR amplicons were commercially Sanger sequenced at Inqaba Biotechnical Industries (Pty) Ltd (Pretoria, South Africa).

### Nucleotide Sequence Identification, MSA, and Phylogenetics

A standard nucleotide BLAST (BLASTn) search was performed for both the tick *AQP1* and 12S gene sequences using NCBI against the non-redundant nucleotide DataBank (Altschul et al., 1990). The tick *AQP1* gene sequences (S1 Data) were translated into 6 open reading frames using ExPASy translate tool (Gasteiger et al., 2003). Protein-protein BLAST algorithm (BLASTp) was used to search for similar tick AQP1 amino acid sequences against PDB using a BLOSUM62 matrix (Berman et al., 2000; Rédei, 2008). Tick AQP1 amino acids and 12S nucleotide sequences from this study and their respective homologous sequences were retrieved the GenBank (Benson et al., 1999; Consortium, 2009). Multiple Sequence Analyses were carried out using MUSCLE (Edgar et al., 2004). Phylogenetic analyses were performed on both datasets using the maximum likelihood method with 1000 bootstrap in MEGA software version X (Tamura et al., 2013). A Pearson correlation matrix of AQP1 homologs was drawn using R studio using Sequir, ggplot2, and reshape2 packages (R Development Core Team, 2010).

### Tick AQP1 Peptides Motif Modeling

Bovine and tick AQP1 amino acid sequences in FASTA format were analyzed in Multiple Em for Motif Elicitation (MEME) tool Version 5.0.5 (Bailey et al., 2009) using the default settings (http://meme-suite.org/tools/meme). This motif-based sequence analysis tool was set to pick at most 50 motifs which appear in at least 2 amino acid sequences. A motif heatmap was generated using a mast text file from MEME and an in-house python script. Tick-specific AQP1 motifs were analyzed for their conservation among different tick species using WebLogo (Crooks et al., 2004).

### Tick AQP1 Protein Homology Modeling

The tick AQP1 homotetrameric homology model was calculated utilizing MODELLER version 9.22 using the “automodel” class (Sali and Blundell, 1993). We used a representative tick AQP1 protein sequence from the study (GenBank: MK334178) as the protein query sequence. AQP1 protein structure templates with sequence identities higher than 30% were selected as suitable templates for homology modeling. A total of 100 homology models were calculated for the tick AQP1 homotetrameric protein. The best four models were selected based on the lowest normalized Discrete Optimized Protein Energy (z-DOPE) score (Eswar et al., 2007). The model structures were evaluated using Protein Structure Analysis (ProSA-web) and Ramachandran plot using RAMPAGE in order to select the best model (Sippl, 1993; Kleywegt and Jones, 1996; Lovell et al., 2003; Wiederstein and Sippl, 2007). The tick-specific AQP1 peptide motifs were mapped onto the tick AQP1 homology model using PyMol, Version 1.7.4 (DeLano, 2002; Forlemu et al., 2017). The structure super-imposition of the tick and bovine AQP1 proteins was done using PyMol (DeLano, 2002).

### *In silico* B Cell Epitope Prediction

The antigenicity of the tick-specific peptide motifs was analyzed semi-empirically based on methods which use physicochemical properties of amino acids residues and their frequencies of occurrence (Chou and Fasman, 1978; Parker et al., 1986; Kolaskar and Tongaonkar, 1990). Antigenicity analyses were completed using the Immune Epitope Database Analysis Resource (IEDB) (http://tools.iedb.org/main/). Seven IEDB methods were used to evaluate the antigenicity of the tick-specific AQP1 peptide motifs:

- BepiPred-1.0 linear epitope prediction which predicts linear B-cell epitopes based on Hidden Markov Models (HMM) and a propensity scale. Residues with a score above a 0.350 default threshold were considered to have a high probability of being part of an epitope (Jespersen et al., 2017)
- BepiPred-2.0: Sequential B-Cell Epitope Predictor which uses a Random Forest algorithm optimized on epitopes and non-epitope amino acids determined from protein crystal structures. Residues with a score above a 0.500 default threshold were considered to have a high probability of being part of an epitope (Jespersen et al., 2017)
- Chou and Fasman Beta-turn prediction which uses the prediction of turns in order to predict epitopes. Residues with a score above a 0.924 threshold were considered to have a high probability of being part of an epitope (Chou and Fasman, 1974)
- Emini surface accessibility scale which is calculated based on surface accessibility. A score >1.000 indicates a high probability of the peptide being found on the protein surface (Emini et al., 1985)
- Karplus and Schulz that determines the flexibility of protein segments based on the B-factors of 31 protein structures. Residues with a score above a 0.962 threshold were considered to have a high probability of being part of an epitope (Karplus and Schulz, 1985)
- Kolaskar and Tongaonkar antigenicity scale which uses a semi-empirical method that is based on the physicochemical probabilities of amino acid residues of the protein of interest and their frequencies of occurrence in experimentally known epitopes from other proteins. Residues with a score above a 1.067 threshold were considered to have a high probability of being part of an epitope (Kolaskar and Tongaonkar, 1990)

Parker-hydrophobicity prediction that relies on a hydrophilic scale based on peptide retention times during high-performance liquid chromatography (HPLC) on a reversed-phase column. Residues above −0.326 threshold were considered to have a high probability of being part of an epitope (Parker et al., 1986).

### Ethical Review

This study was approved by the Makerere University, School of Biosecurity, Biotechnology and Laboratory Sciences Research and Ethics Committee (SBLS/REC/16/136) and Uganda National Council for Science and Technology (A 513). Informed consent was obtained from cattle owners before their cattle were enrolled into the study. All efforts were made to minimize animal stress during cattle restraint and tick collection. These activities were conducted by qualified veterinarians.

## Results

The amino acid sequence numbering used in this section is based on tick AQP1 (Accession: MK334178).

### Tick Species Were Confirmed Using 12S rRNA Gene Sequencing

**Figure 4:**
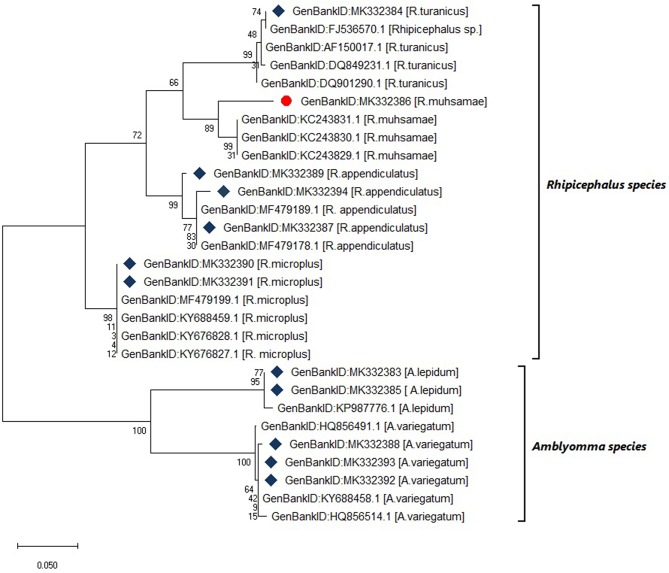
Maximum likelihood phylogenetic tree of tick species based on 12S rDNA gene sequences. Key: 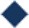 indicates **12S rDNA gene** sequences from this study 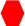 and indicates a tick species identified for the first time in Uganda by this study. The phylogenetic tree was constructed using MEGA X software with the maximum likelihood model with 1000 bootstrap. The scale indicates the rate of nucleotide substitution per node.

Six tick species were morphologically identified and confirmed by 12S rRNA gene sequencing. The 12S rRNA gene sequences formed two distinct clusters with GenBank sequences of the genera *Rhipicephalus* and *Amblyomma* (Figure 4). The tick species included *Rhipicephalus turanicus, Rhipicephalus muhsamae*, and *Amblyomma lepidum* for Kotido district isolates. *Rhipicephalus appendiculatus* for Mbarara, Serere, and Kiruhuura district isolates. *Rhipicephalus microplus* and *Amblyomma Variegatum* for Serere district isolates.

### Diversity of Tick AQP1 Protein Sequences and Orthologs

**Figure 5:**
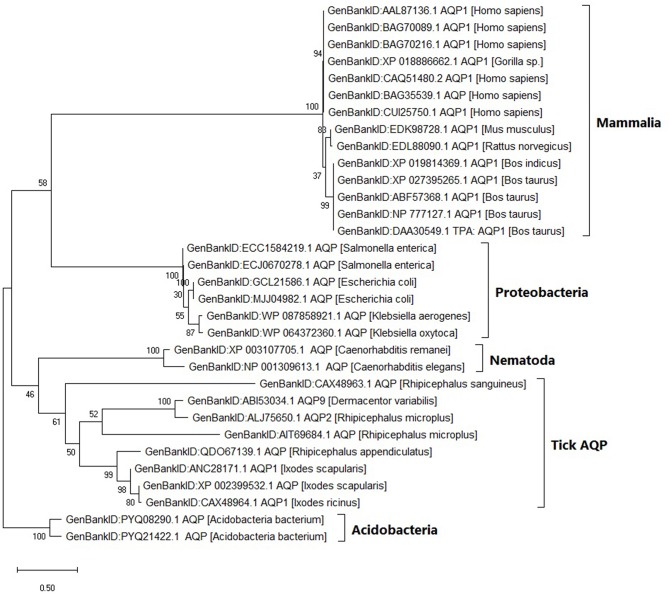
Maximum likelihood phylogenetic tree of AQP1 amino acid sequences from various tick species and other organisms. The tree is drawn to scale, with branch lengths measured in the number of substitutions per site. This analysis involved 32 amino acid sequences. There were a total of 346 positions in the final dataset. Evolutionary analyses were conducted in MEGA X.

Within the tick AQP1 amino acid sequences, we observed substitution rates ranging between 0.025 and 0.800. Amino acid substitution rates close to 0 indicate high sequence similarity. In contrast, a comparison of ortholog AQP1 and tick AQP1 amino acid sequences showed higher substitution rates ranging between 1.110 and 1.270. The tick AQP1 amino acid sequences grouped in a distinct clade separate from other orthologous AQP1 amino acid sequences (Figure 5).

**Figure 6:**
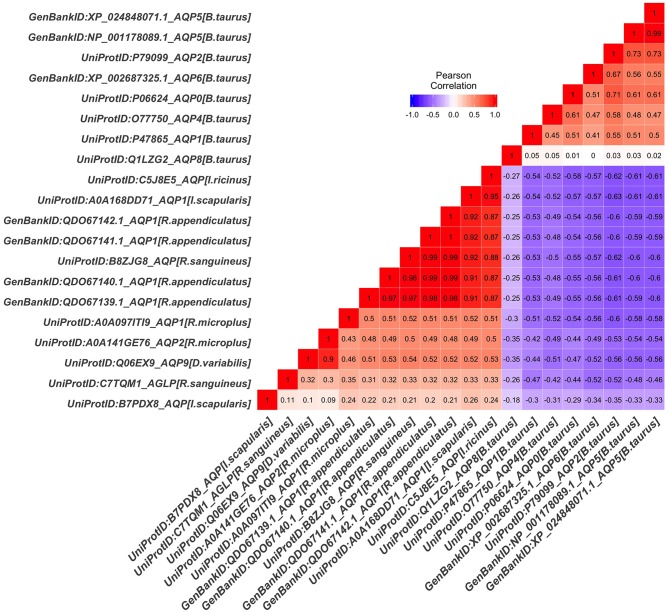
Protein dissimilarity matrix of tick AQP1 amino acid sequences and their orthologs. Blue and red color ranges denote negative and positive correlation respectively The function scale fill gradient2 was used with the argument limit, c (−1,1).

The tick AQP1 amino acid sequences and their orthologs were further analyzed using a protein dissimilarity matrix that showed that the tick AQP1 amino acid sequences had positive Pearson correlation coefficients (r) ranging from 0.160 to 1.000 (Figure 6). Tick AQP1 amino acid sequences were negatively correlated to those of bovines and humans (r = −0.4 to −0.6) (Figure 6). Altogether, the phylogenetic and Pearson correlation matrix analyses show that much as AQP1 is conserved among different species, some differences exist between tick AQP1 and bovine AQP1 protein sequences.

### Tick and Bovine Peptide Motif Modeling

**Figure 7:**
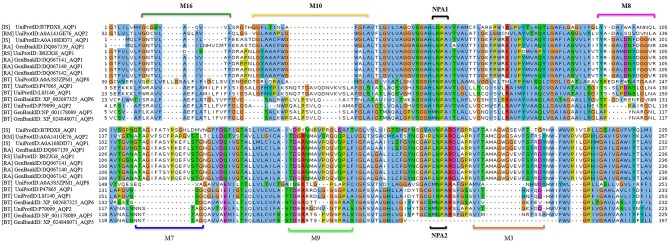
Multiple Sequence Alignment (MSA) of tick and bovine AQP1 amino acids. The NPA1 and NPA2 motifs were conserved among the AQP1 orthologs. Also shown are the tick specific peptide motifs: M3, M7, M8, M9, M10, and M16. The MSA was completed using MUSCLE.

MSA analysis of the tick AQP1 and its bovine orthologs showed local regions with high sequence conservation. The Asparagine-Proline-Alanine (NPA) motif (NPA1: residues 49-51, NPA2: residues 180-181) was conserved in all amino acid sequence orthologs. The NPA is a highly conserved hydrophobic motif that forms part of the pore for each AQP1 monomer (Finn and Cerda, 2015). In addition, tick AQP1 motifs contained an aspartic acid (D) residue after the second NPA motif; a signature for aquaglyceroporins (Figure 7).

**Figure 8:**
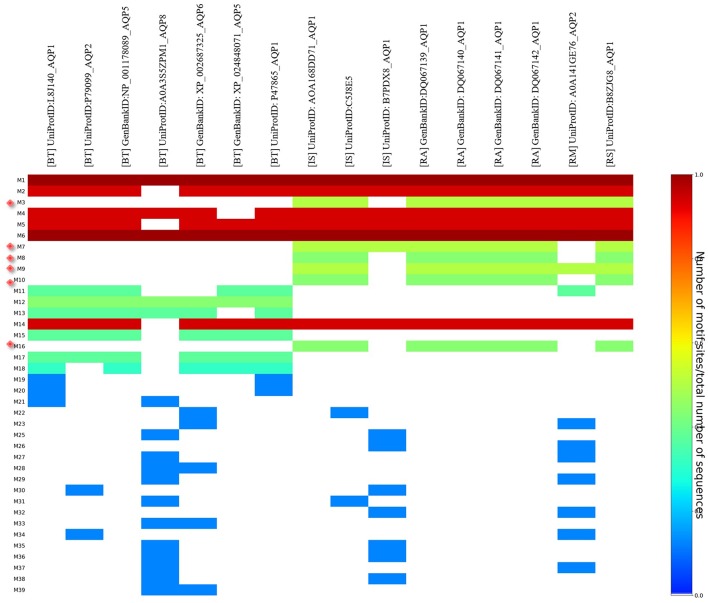
Heat map of tick and bovine AQP1 peptide motifs. Tick specific AQP1 peptide motifs: M3, M7, M8, M9, M10, and M16 are denoted by red diamonds. Six AQP1 peptide motifs; M1, M6, M2, M4, M5, and M14 were common to both tick and bovine AQP1. Key: [BT] stands for *B. taurus*, [IS] stands or *I. scapularis*, [RA] stands for *R. appendiculatus*, [RS] stands for *R. sanguineus* and [RM] stands for *R. microplus*.

MEME analysis was used to identify tick-specific AQP1 peptide motifs. The tick-specific AQP1 motifs were conserved among the four different tick species (*Ixodes scapularis, R. appendiculatus, R. sanguineus*, and *R. microplus*) with E-values ranging from 1.6e-108 to 7.6e-013. These motifs included: M3 (residues 193-212), M7 (residues 106-125), M8 (residues 85-104), M9 (residues 149-168), M10 (residues 12-31), and M16 (residues 2-11) (Figure 8).

**Figure 9:**
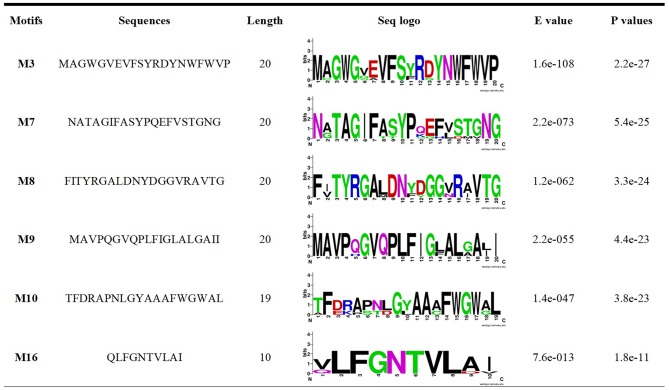
Amino acid and SeqLogo-sequences of tick specific AQP1 peptide motifs.

The position *p*-value (the probability of a single motif appearing in the observed consensus sequence) of the consensus tick AQP1 sequence (MK334178) further showed this conservation level across all the six motifs. Position *p-*values <0.0001 were considered significant. All the tick-specific AQP1 peptide motifs had lengths ≥ 19 amino acid residues except M16 whose length was 10 amino acid residues. The motif conservation level was biased to amino acids with similar physicochemical properties. For instance, motifs M3, M9, M10 consisted of a high number of conserved hydrophobic amino acids (residues 16–20, 9–20, and 11–19 respectively) which are colored in black. The most highly conserved motif was M16 with over 70% of its residues being hydrophobic. Most of this motif was located in the transmembrane domain. Motif M7 contained a high number of substitutions all of which were observed in one tick species (*I. scapularis: NCBI* nucleotide sequence XP002399532) with a *p*-value of 5.4e-25. Motifs M7 and M8 were largely made up of hydrophilic residues (Figure 9).

### Tick AQP1 Homology Modeling and Structure Analysis

Two *Escherichia coli* strain K12 AQP1 structures, namely 1LDF and 1FX8 were selected as template structures from PDB after a sequence similarity search. Homology model04 which had a z-DOPE score of −0.358 and a ProSA z-score of −3.400 was selected for use in further analyses (S12 Figure). A z-DOPE score equal to or < -1 indicates that the protein model is close to the native structure (Bastianelli et al., 2011) (Table 1). A Ramachandran plot analysis using RAMPAGE showed that model04 had 884 (92.9 %) of its residues in the favored region, 45 (4.7 %) in the allowed region and 23 (2.4 %) in the outlier region (Table 1). See alignment file (PIR) and atomic coordinates of the top four homology models generated in S2–S6 Data.

**Table 1:** Comparison of tick AQP1 homology models and template AQP1 structures.

		**RMSD**	
**Model name**	**z-DOPE score**	**1FX8**	**1LDF**
Model04	−0.358	0.408	0.615
Model03	−0.346	0.375	0.570
Model02	−0.344	0.391	0.578
Model01	−0.332	0.394	0.573

*The homology model with a z-DOPE score close to −1 indicate homology of good quality. For the structure super-imposition of the homotetramers RMSD values close to zero indicated that the two protein structures are structurally similar (S6 Data, S7–S9 Figures)*.

**Figure 10:**
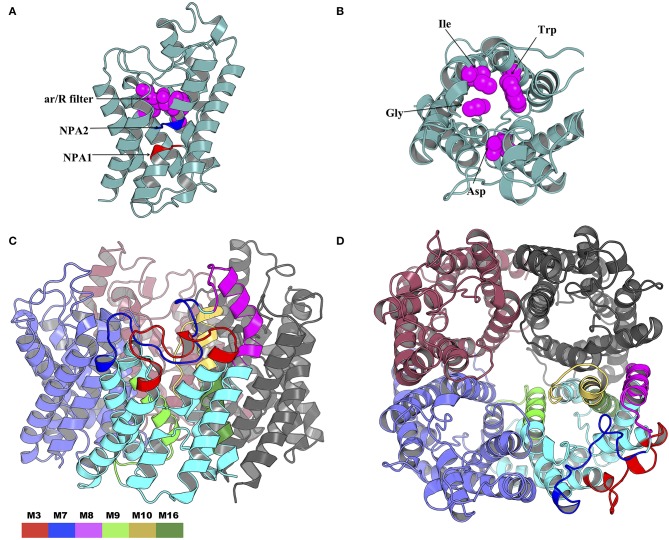
Cartoon rendering of the tick AQP1 homology model and motif mapping. **(A)** A side-view of the modeled tick AQP1 monomer showing the position of the two Asparagine-Proline-Alanine (NPA) motifs and the aromatic-arginine (ar/R) filter. **(B)** A top view of the modeled tick AQP1 monomer showing the amino acid sequences that constitute the ar/R filter (chain A residues: Trp33, Gly136, Ile171, and Asp188). **(C)** A cartoon rendering of the tick AQP1 protein and **(D)**. A surface view rendering of the tick AQP1 protein. Both **(C,D)** show the mapped tick-specific peptide motifs. Motifs M7 and M8 were located on the protein surface and in the extra-cellular domain while M9, M10, and M6 were located in the transmembrane domain. The motifs were colored as follows: M3- red, M7- blue, M8- purple, M9- light green, M10- yellow, and M16- dark green.

The NPA1 (chain A residues 49–51) and NPA2 (chain A residues 180–181) motifs are located within the transmembrane domain (Figure 10A). The aromatic-arginine (ar/R) filter was made of residues Trp33, Gly136, Ile171, and Asp188 (Figure 10B). Mapping of the six motifs onto the modeled tick AQP1 protein structure showed that motifs M9, M10, and M16 are located within the transmembrane protein domain while motifs M3, M7, and M8 are located in the extracellular protein domain (Figures 10C,D). Motif M10 was more localized in the inner part of the homotetrameric protein while motif M3, M7, and M8 were located on the extracellular surface of the quaternary structure of the AQP1 protein model (Figures 10C,D). The structural superimposition of the tick AQP1 homology model04 and bovine AQP1 (PDB: 1J4N) showed that the proteins were structurally similar with an RMSD value of 3.269 for the homotetrameric structures and RMSD = 1.475 for similar chain monomers (S13 Figure). The structural similarities were mainly observed in the transmembrane domain while variations were observed in the loops located on the extracellular and cytoplasmic domains of the protein (S13 Figure).

### *In silico* Analysis of the B Cell Peptide Motif Antigenicity

**Figure 11:**
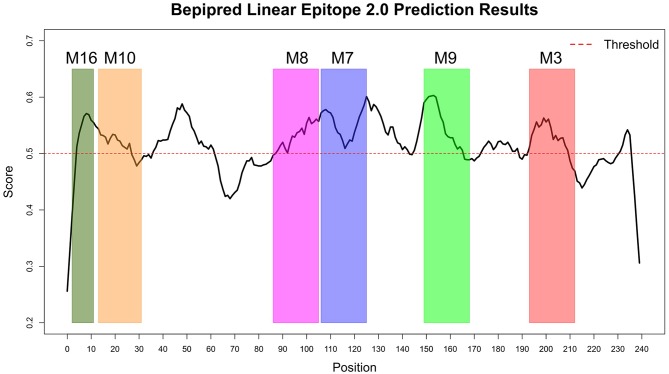
BepiPred 2.0 Linear Epitope Prediction method. All the motif did show potential of being part of the epitope, however motif M7 and M8 were the only motifs with all the residues above the threshold (0.5). Residues with scores above this threshold were considered to have a high probability of being part of an epitope.

**Figure 12:**
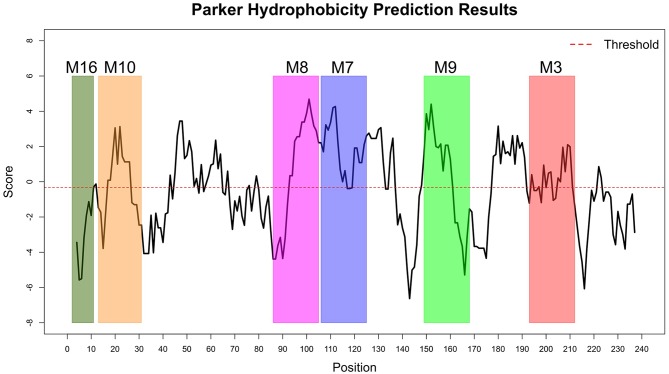
Parker Hydrophilicity Prediction methods. Motif M7, C terminal end of M8, N terminal end of M9, part of M9 and M10 were potentially part of the epitopes with residue scored above the threshold (-0.326). However, motif M16 residues-scores were below the threshold level. Residues with scores above threshold were considered to have a high probability of being part of an epitope.

The Immune Epitope Database (IEDB) methods were used to evaluate whether the motifs were located in potential B cell epitopes. The results indicate that motifs M7 and M8 might be potential antigenic peptide epitopes. Motifs M7 and M8 had Mean BepiPred 2.0 Linear Epitope Prediction scores 0.551 and 0.528 that were above the 0.5 threshold value (Figure 11 and Table 2). Motifs M7 and M8 also had mean Parker hydrophilicity scores of 1.784 and 1.536, respectively, that were above the −0.326-threshold score (Figure 12). The two motifs also showed mean Chou and Fasman Beta-Turn Predictions and Karplus and Schulz Flexibility predictions that were slightly above their respective thresholds Motif M7 had a mean BepiPred 1.0 Linear Epitope Prediction of 0.464 that was above the 0.350 threshold value unlike motif M8 that had a mean score of −0.494. Motif M16 which was the shortest peptide motif (Figure 9) and located in the transmembrane region of the protein (Figure 10) was below the thresholds for most of the methods used indicating it was a poor epitope candidate. Other transmembrane peptide motifs such as M9 and M10 were also predicted to be poor epitope candidates (Table 2). Extra supporting information on B cell peptide motif antigenicity has been provided as S7–S11 Figures.

**Table 2:** Prediction of B cell peptide motif antigenicity using IEDB Analysis Resource.

**Motifs**	**Mean BepiPred 1.0 (0.350)**	**Mean BepiPred 2.0 (0.500)**	**Mean Chou and Fasman (0.924)**	**Mean Emini (1.000)**	**Mean Karplus (0.962)**	**Mean Kolaskar (1.067)**	**Mean Parker (−0.326)**
M3	−0.285	0.529	0.971	1.742	0.937	1.022	−0.396
M7	0.464	0.551	1.003	1.370	0.997	1.021	1.784
M8	−0.494	0.528	1.028	1.340	0.989	1.029	1.536
M9	−0.100	0.550	0.911	1.088	0.977	1.087	−0.559
M10	−0.278	0.518	0.946	1.143	0.952	1.021	−0.476
M16	−1.760	0.502	0.811	0.373	0.962	1.115	−2.284

*Values in parenthesis represent threshold scores; motifs with scores above the threshold values are considered to have a high probability of being part of B cell epitopes*.

## Discussion

In this study, a combination of DNA sequencing and *in silico-*based methods were used to predict tick-specific AQP1 peptide motifs. This involved comparing tick AQP1 protein sequences isolated from four districts in Uganda and AQP1 ortholog sequences from GenBank and PDB. We identified a total of 6 tick-specific AQP1 peptide motifs that were mapped onto the tick AQP1 protein structure (homology model). The predicted motifs were also assessed for their potential of being candidate peptide epitopes using IEDB tools.

The study-tick AQP1 protein sequences showed regions of similarity with AQP1 orthologs (Figure 7). This is due to the fact that AQP1 plays similar functions in the different species. In addition, all AQP proteins have 6 transmembrane alpha helical domains, a signature characteristic to all Major Intrinsic Protein (MIP) superfamily proteins (Finn and Cerda, 2015). The highest AQP1 sequence similarity within the AQP1 orthologs was observed mainly around the Asparagine-Proline-Aline (NPA) motif and aromatic/Arginine (ar/R) selectivity filters (Beitz et al., 2006; Gonen and Walz, 2006; Yakata et al., 2007; Figure 7). These filters slow down the flow of molecules across the protein pore by a Grotthuss mechanism (Beitz et al., 2006; Gonen and Walz, 2006; Yakata et al., 2007). Similar studies have indicated that the arrangement of the ar/R residues directly correlates with the functional properties of the channel (Beitz et al., 2006; Gonen and Walz, 2006; Yakata et al., 2007). Classical water-selective aquaporins (CAQPs) such as AQP1 usually show tight ar/R clusters within which passage of water is allowed while ions and glycerol are blocked (Yakata et al., 2007; Finn and Cerda, 2015; Ishibashi et al., 2017) (Figures 10A,B). The tick AQP1 from this study contained an aspartic acid residue (D) after the second NPA motif which is the signature sequence of Aquaglyceroporin (AQGP) (Figures 7, 10A,B). This finding is in conformity with previous studies which looked at AQP1 proteins from different tick species (Bowman and Sauer, 2004; Guerrero et al., 2014; Évora et al., 2017; Ishibashi et al., 2017). An aspartic acid residue always coexists with a longer loop which increases the pore's permeability to larger molecules such as glycerol (Ishibashi et al., 2011). Despite the presence of a D residue, this functional characterization of tick AQP1 does not warrant transportation of glycerol or urea (Ball et al., 2009). Such tight water transport mechanisms seen in tick AQP1 helps them to concentrate the blood meal component in the midgut by removing only water back into the host via the salivary gland (Bowman and Sauer, 2004).

The high sequence similarity within the AQP1 from all different tick species further emphasizes its potential for use as a catch-all anti-tick vaccine candidate (Contreras and de la Fuente, 2017). Whole tick AQP1 structure super-imposition to the bovine AQP1 protein indicated that the two proteins were structurally similar (RMSD = 3.269 for the homotetramers and RMSD = 1.475 for similar chain monomers). Such similarities to the host protein can affect vaccine efficacy and potentially cause adverse autoimmune effects if vaccinated with the whole AQP1 recombinant protein (Overwijk et al., 1999). This challenge could have been the reason for failure of the recombinant AQP1 protein as vaccine against *I. ricinus* (Campbell et al., 2010). Future AQP1-anti-tick vaccines therefore should at least be based on reverse vaccinology methods focusing on tick-specific AQP1 peptide motifs that might constitute vaccine development pipeline (Article, 2016). Peptide based vaccines use specific peptide fragments that induce high specific immune responses, thereby increasing vaccine efficacy and reducing potential adverse effects caused by whole protein vaccination (Li et al., 2014). A number of peptide-derived vaccines have been developed, some of these are in clinical trials. A good example is the discovery of neutralizing epitopes to HIV and influenza viruses (Liu et al., 2007; Sui et al., 2009).

MSA of tick AQP1 amino acid sequences and their bovine orthologs highlighted some differences between them (Figure 7). These regions of difference were observed in the loops, particularly those located in the extracellular domain (S13 Figure). Structural mapping of peptide motifs onto the AQP1 homology model protein showed that motifs M9, M16, part of M10 and M3 were all located on tick AQP1 transmembrane domains (Figure 10). Motif modeling and sequence logo identification showed that these motifs contained high number of hydrophobic residues which usually encode for most transmembrane alpha helices (Sonnhammer and Krogh, 1998). This phenomenon is due to the fact that hydrophobic residues are non-polar, thus, they have to fold in such a way that they are buried in the lipid bilayer of the cell membrane (Sonnhammer and Krogh, 1998). Moreover, these motifs (M3, M9, M10, and M16) showed relatively lower potential of being B cell epitope region compared to motifs M7 and M8 (Table 2).

Both motif M7 and M8 were linear epitopes, yet most B cell peptide motif are discontinuous in nature. Nevertheless continuous epitopes can evoke antibodies that can recognize and bind to antigenic proteins, albeit with lower binding affinities (Chen et al., 2009). The motifs identified by this study require further evaluation using wet-lab animal models and *in vitro* tests in order to determine their immunogenicity.

## Conclusion

We identified two motifs (assigned names M7 and M8) that can potentially be incorporated into AQP1 peptide anti-tick vaccine development efforts. The peptide motifs were highly conserved among tick AQP1 and differed from bovine and human AQP1. This indicates that they are an interesting potential targets against a wide range of tick species. We envisage future studies where we can apply the approaches used in this study on the entire tick proteome. This will enable us to identify a larger set of potentially antigenic peptides. We also require wet-lab *in vivo* and *in vitro* studies to determine the immunogenicity and efficacy of the tick-specific M7 and M8 peptide motifs. The wet-lab studies can also help determine the conformations the peptides will take in vaccine formulations and the possibility of cross-reactivity with other bovine proteins.

## Data Availability Statement

The datasets generated for this study can be found in the NCBI data base under the following accession numbers: MK332384, MK332386, MK332389, MK332394, MK332387, MK332390, MK332391, MK332383, MK332385, MK332388, MK332393, MK332392, MK334175, MK334178, MK334176, MK334177.

## Ethics Statement

The animal study was reviewed and approved by Makerere University, School of Biosecurity, Biotechnology and Laboratory Sciences Research and Ethics Committee (SBLS/REC/16/136) and Uganda National Council for Science and Technology (A 513). Written informed consent was obtained from the owners for the participation of their animals in this study.

## Author's Note

Author Magambo Phillip Kimuda is a member of the Trypanogen Consortium (www.trypanogen.net).

## Author Contributions

CN and DM designed and implemented the study. CN, DM, JN, WA, FM, RT, SO, and MK performed the experiments and data analysis. All authors participated in writing and proofreading the manuscript and approved the final manuscript for publication.

### Conflict of Interest

The authors declare that the research was conducted in the absence of any commercial or financial relationships that could be construed as a potential conflict of interest.

## Supplementary Material

S1 Data Fasta file of tick AQP1 sequences MK334175- MK334178 (*R. appendiculatus*).

S2 Data PIR alignment file of MK334178 query sequence and templates (1FX8 and 1LDF).

S3-6 Data Atom coordinate files for tick AQP1 homology models (model01-model04).

S7 Figure Chou and Fasman Beta-turn Prediction method. Motif M7, M8, and M9 had the high number of residues above the threshold score (0.924). While Motif M16 had no residues above the threshold. Residues with scores above threshold were considered to have a high probability of being part of an epitope.

S8 Figure Karplus and Schulza Flexibility Prediction method. All the Motif had high residues above the threshold (1) except M3 and M16 which had higher residues below the threshold. Residues with scores above threshold were considered to have a high probability of being part of an epitope.

S9 Figure Bepipred 1.0 Linear Epitope Prediction method. Motif M7 and the C-terminal end of M8 shown potential of being part of the peptide with antigenicity score above the threshold level (0.35). residues with scores above threshold were considered to have a high probability of being part of an epitope.

S10 Figure Emini Surface Accessibility Prediction method. All the motif except motif M16 had high number of residues above the threshold score (0.924). Residues with scores above threshold were considered to have a high probability of being part of an epitope.

S11 Figure Kolaskar and Tongaonkar Antigenicity Prediction method. Motif M16 followed by part of motif M10 and M8 at the C terminal end had values above the threshold (−0.326). Motif M7, M9, and M3 had a high number of residues below the threshold. Residues with scores above threshold were considered to have a high probability of being part of an epitope.

S12 Figure Evaluation of the top tick AQP1 homology model (model04) using ProSA. **(A)** Model04 had a ProSA z-score of−3.400 that clustered well with experimentally determined models, and **(B)** A plot of residue scores of model04.

S13 Figure Structural superimposition of tick AQP1 homology model and bovine AQP1 (PDB: 1J4N). Structural superimposition of homotetrameric structures gave an RMSD = 3.269 while superimposition of a single chain monomer gave an RMSD = 1.475.
