# Supplementary figures and images for: Identification of Ixodid Tick-Specific Aquaporin-1 Potential Anti-tick Vaccine Epitopes: An in-silico Analysis
Source: Front Bioeng Biotechnol. 2019 Sep 26;7:236. doi: 10.3389/fbioe.2019.00236 (PMC6775757; doi:10.3389/fbioe.2019.00236)

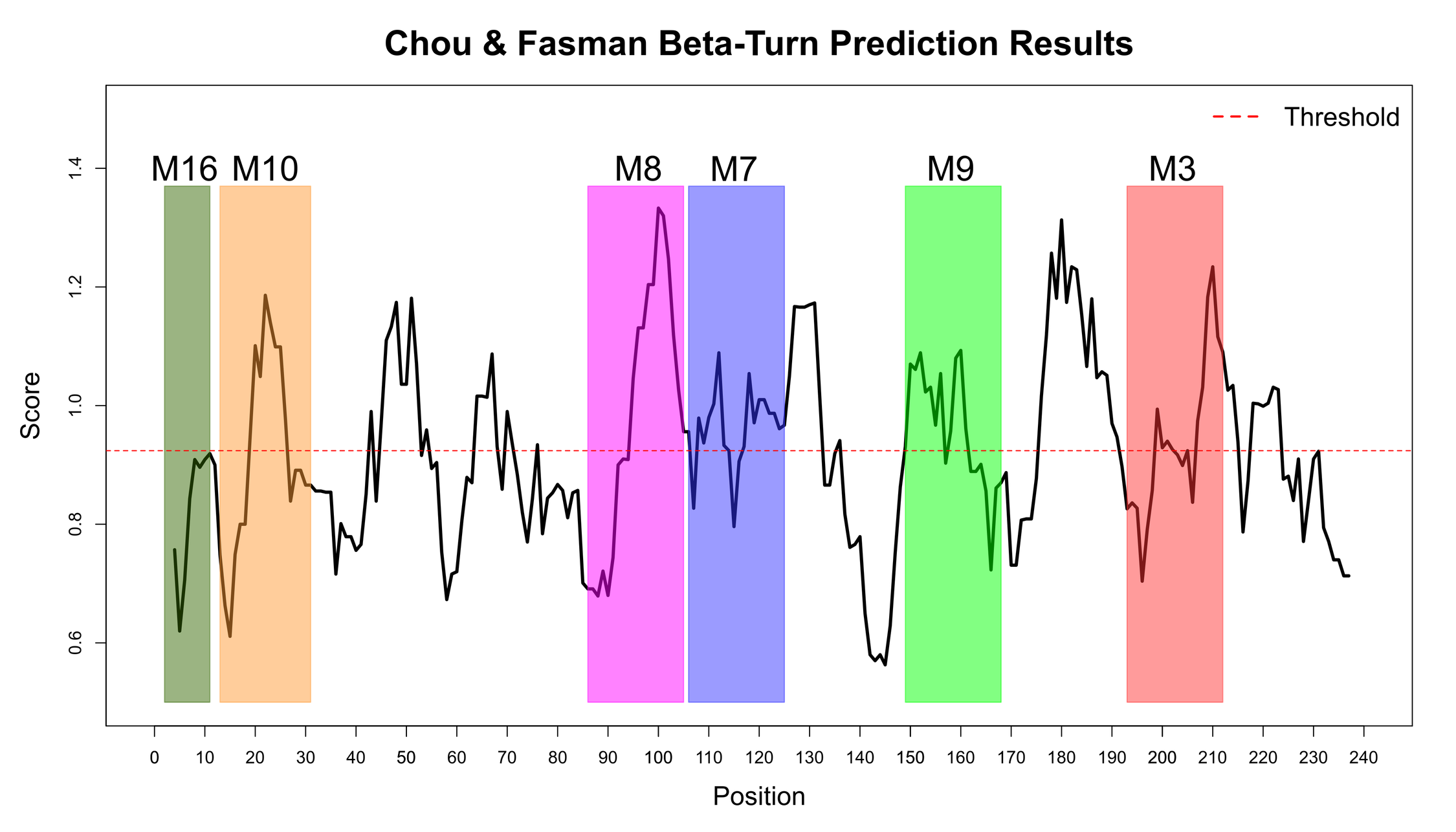

Supplement: S1 Data — Fasta file of tick AQP1 sequences MK334175- MK334178 (R. appendiculatus). [file Data_Sheet_1.ZIP › Supplementary files/S7 Figure.tif]

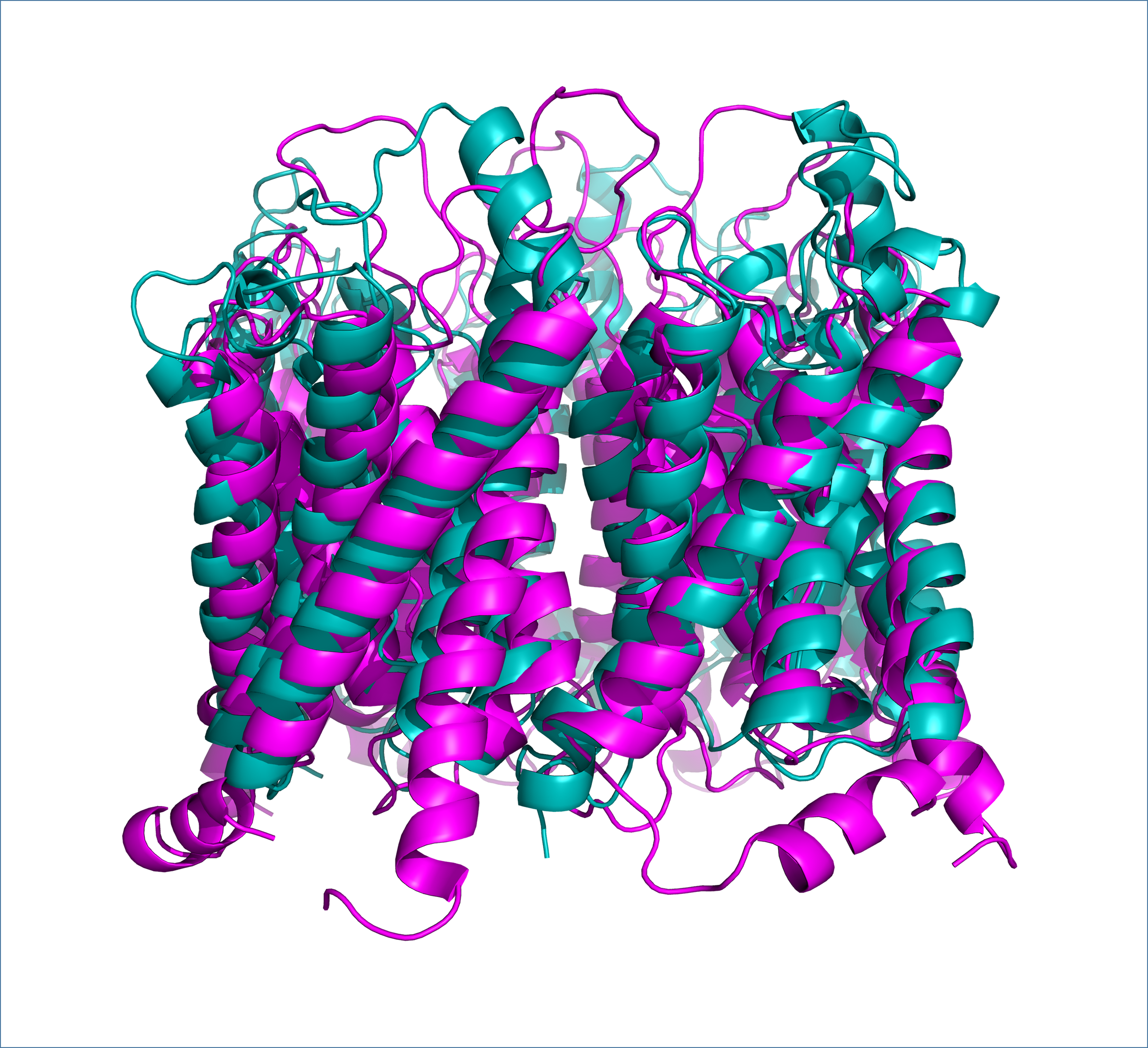

Supplement: S1 Data — Fasta file of tick AQP1 sequences MK334175- MK334178 (R. appendiculatus). [file Data_Sheet_1.ZIP › Supplementary files/S13 Figure.tif]

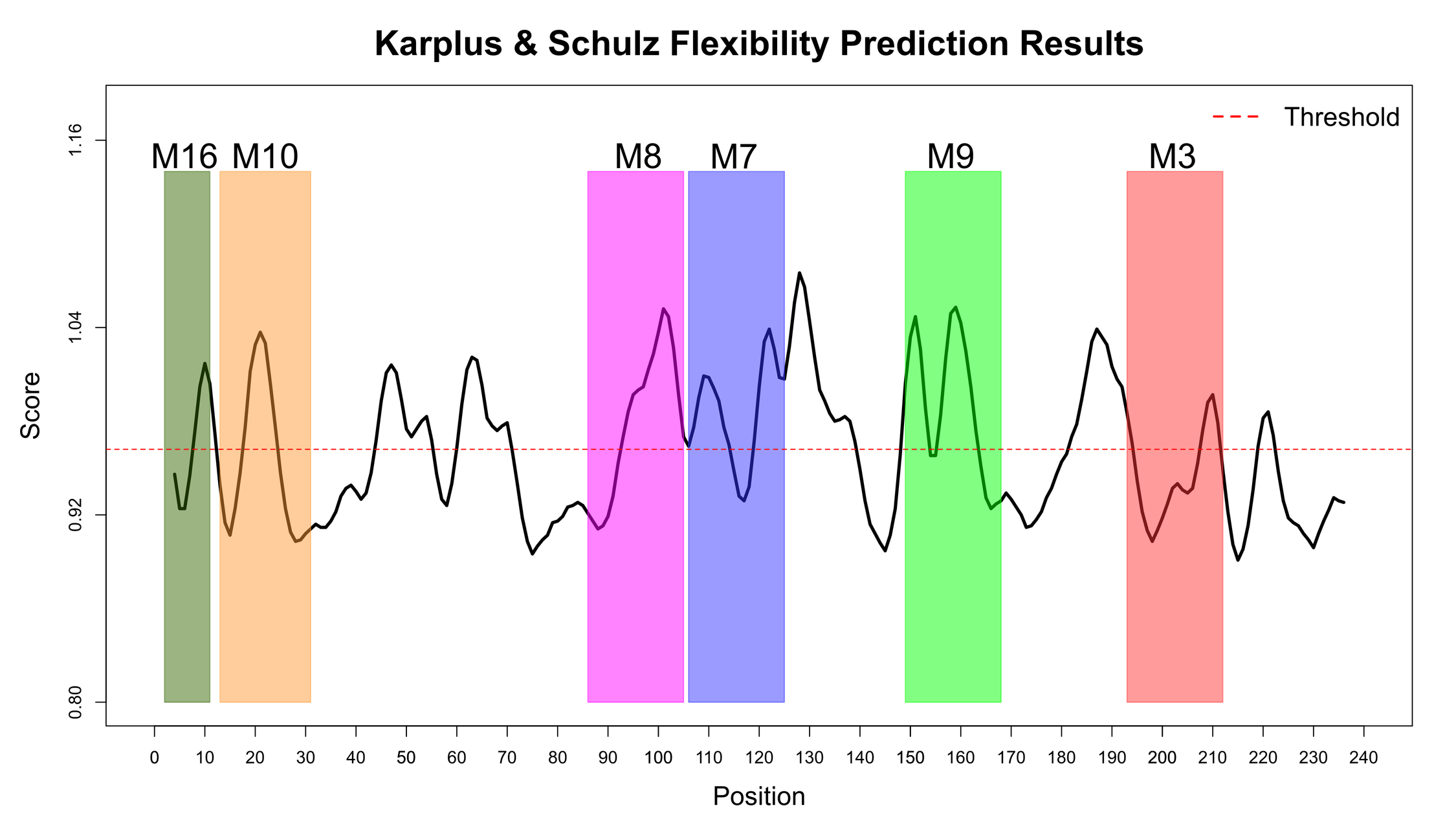

Supplement: S1 Data — Fasta file of tick AQP1 sequences MK334175- MK334178 (R. appendiculatus). [file Data_Sheet_1.ZIP › Supplementary files/S8 Figure.tif]

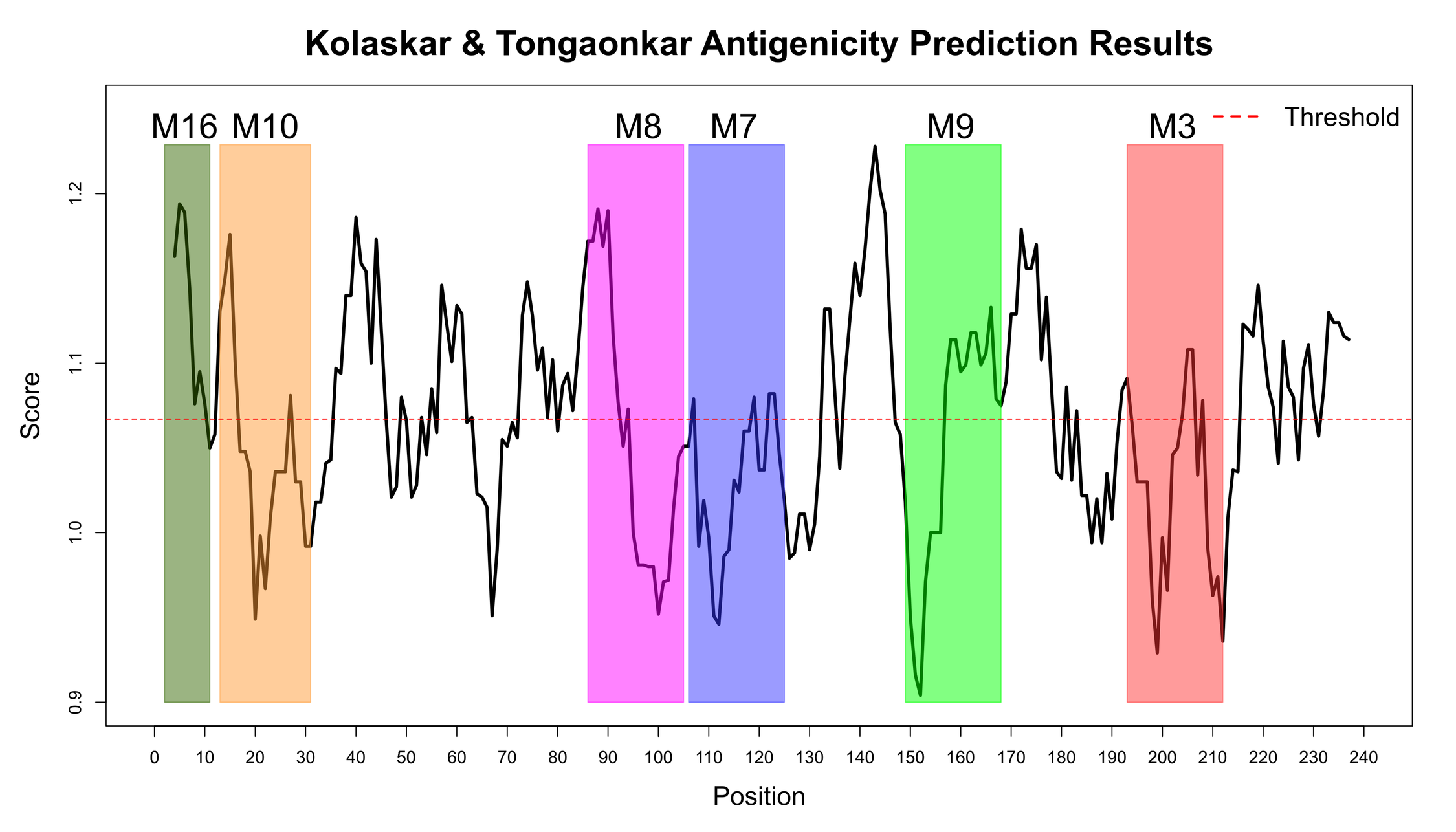

Supplement: S1 Data — Fasta file of tick AQP1 sequences MK334175- MK334178 (R. appendiculatus). [file Data_Sheet_1.ZIP › Supplementary files/S11 Figure.tif]

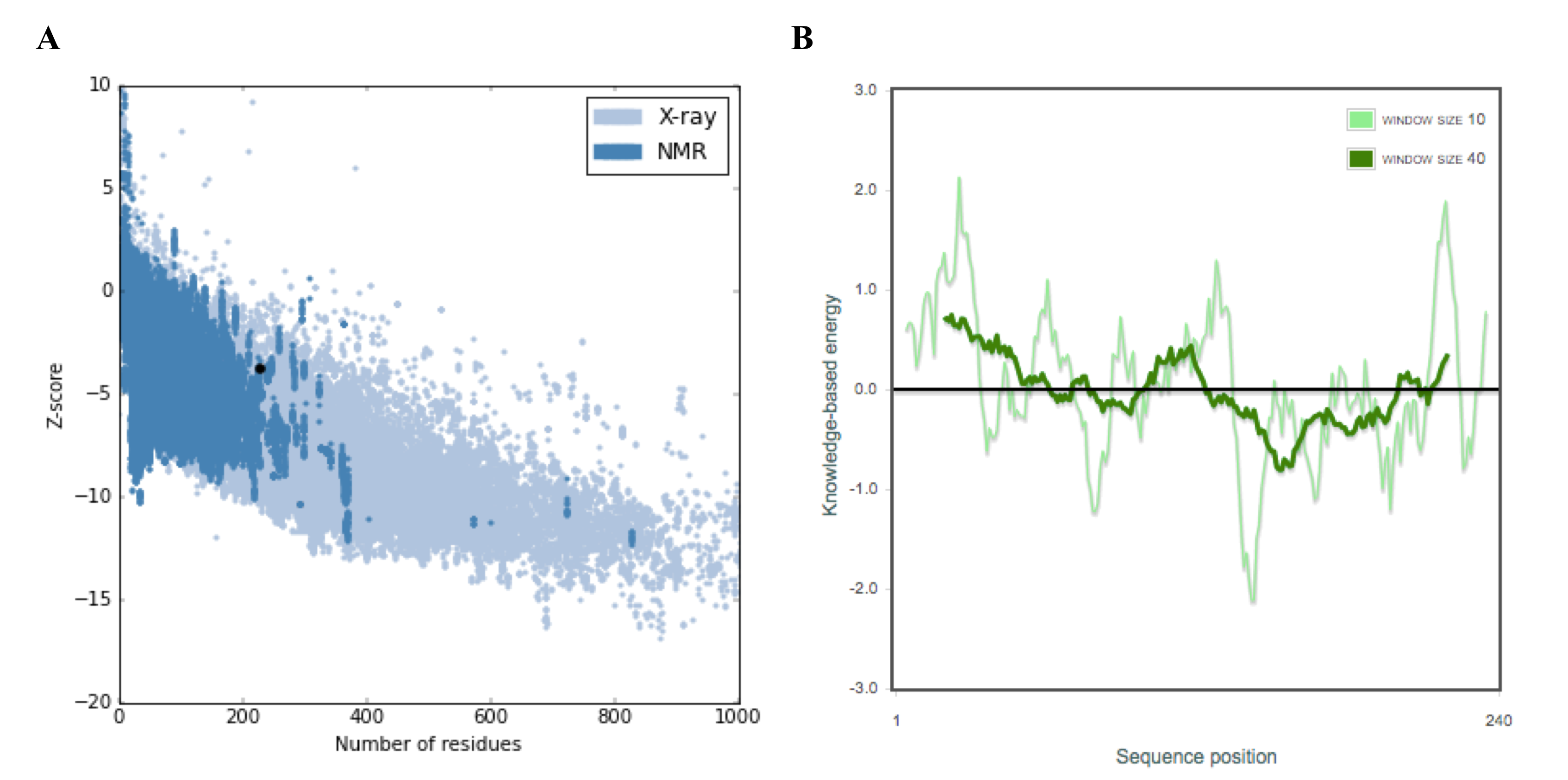

Supplement: S1 Data — Fasta file of tick AQP1 sequences MK334175- MK334178 (R. appendiculatus). [file Data_Sheet_1.ZIP › Supplementary files/S12 Figure.tif]

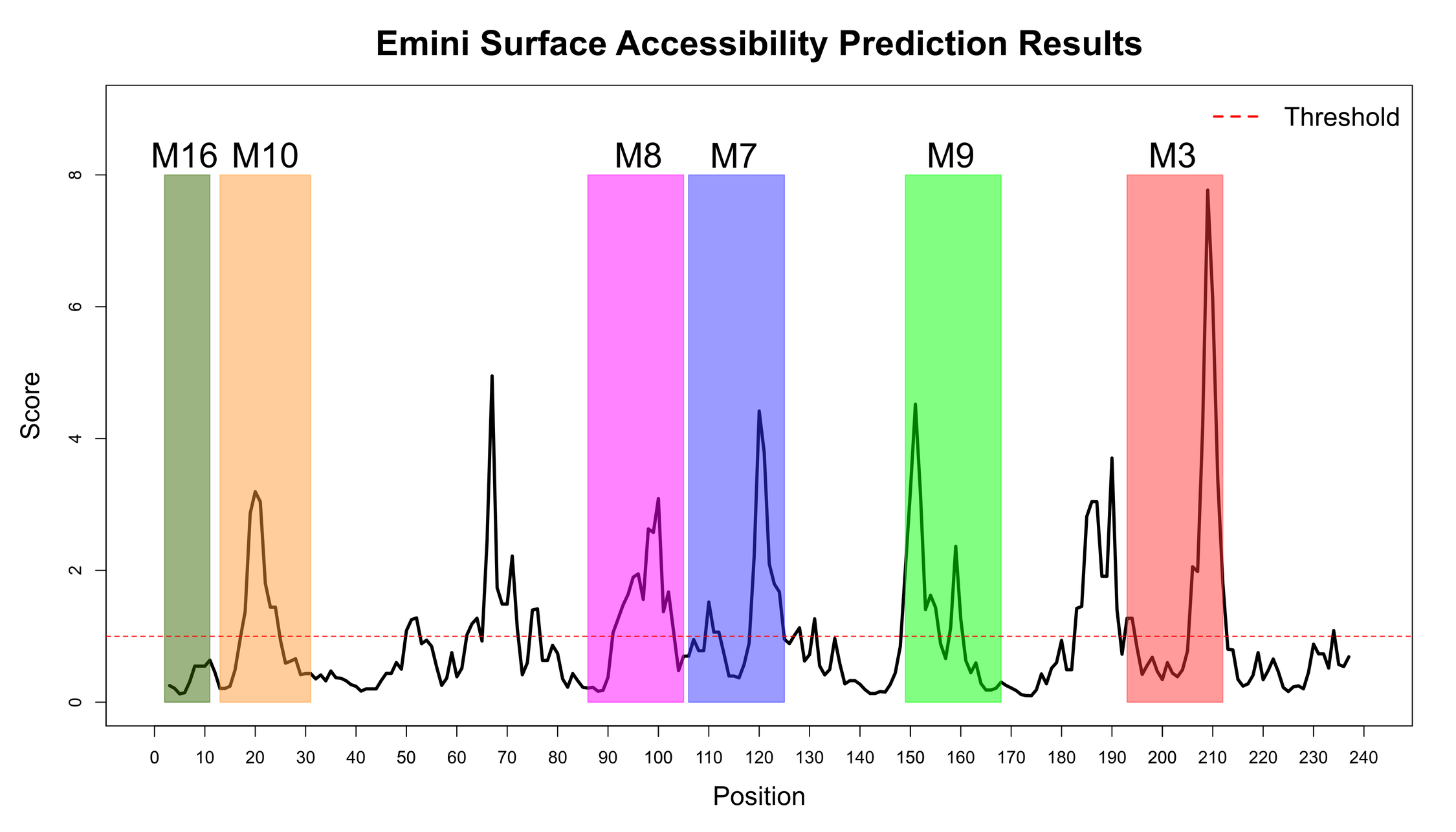

Supplement: S1 Data — Fasta file of tick AQP1 sequences MK334175- MK334178 (R. appendiculatus). [file Data_Sheet_1.ZIP › Supplementary files/S10 Figure.tif]

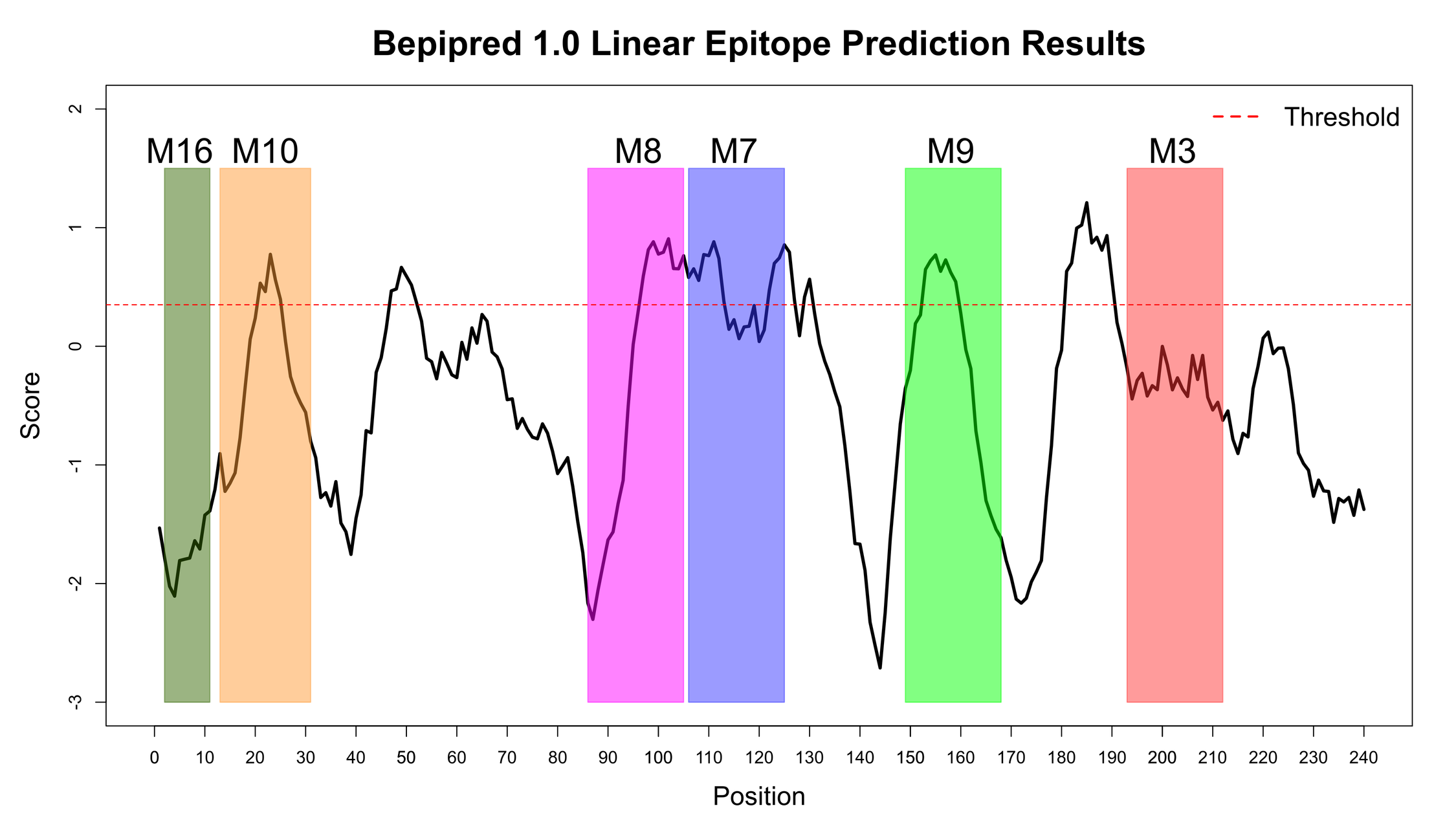

Supplement: S1 Data — Fasta file of tick AQP1 sequences MK334175- MK334178 (R. appendiculatus). [file Data_Sheet_1.ZIP › Supplementary files/S9 Figure.tif]
